# Varietal Differences in the Environmental Behavior of ^14^C-Caffeine in Tea Plants: Accumulation, Subcellular Distribution, and Metabolism

**DOI:** 10.3390/biology14020177

**Published:** 2025-02-10

**Authors:** Yan Chen, Kaitai Song, Huizhong Hu, Haiyan Wang, Xinqiang Zheng

**Affiliations:** Key Laboratory of Nuclear Agricultural Sciences of Ministry of Agriculture, College of Agriculture and Biotechnology, Zhejiang University, Hangzhou 310058, China

**Keywords:** ^14^C-caffeine, tea seedlings, subcellular distribution, targeted accumulation, metabolism

## Abstract

Caffeine contamination in water sources raises concerns about its transfer to agricultural products and potential risks to human health through the food chain. Despite these concerns, limited research has focused on the accumulation and distribution of exogenous caffeine in tea plants. This study explored the uptake, translocation, targeted accumulation, subcellular distribution, and preliminary metabolism of ^14^C-labeled caffeine in a hydroponic tea seedling system. After 192 h of cultivation, more than 83.8% of the caffeine had been removed from the nutrient solution. Within the plants, ^14^C-caffeine and its metabolites predominantly accumulated in the roots. Subcellular analysis indicates that in root cells, ^14^C was mainly distributed in the soluble fraction, cell walls, and plastids, while in shoot cells, it was concentrated in the soluble fraction and cell walls. Metabolic profiling reveals distinct varietal differences: in Longjing 43 tea seedlings, ^14^C was predominantly present as the caffeine parent compound, whereas in Jiaming No. 1 tea seedlings, ^14^C was found both as the parent compound and as its metabolite, xanthine. This study revealed differences in the uptake, translocation, and metabolism of exogenous caffeine among different tea plant varieties, providing broader insights into the impact of caffeine pollution on agricultural ecosystems.

## Simple Summary

Caffeine contamination in water sources is a growing concern due to its potential impact on food safety and human health. This study focused on understanding how caffeine from water sources accumulates in tea plants, specifically how it moves within the plant and its distribution at the cellular level. Using a hydroponic system, we tracked caffeine labeled with a radioactive isotope ^14^C. We found that the caffeine mainly accumulated in the roots, with specific distribution patterns observed within the cells. The study also discovered that different varieties of tea plants metabolite caffeine differently. In one variety, the caffeine remained mostly unchanged, while in another variety, caffeine was converted into xanthine.

## 1. Introduction

Caffeine, a novel organic contaminant, has been detected in surface waters and soils worldwide, with concentrations ranging from ng/L to mg/L, making it a reliable indicator of water pollution [1,2,3]. In Biscayne Bay, Florida, caffeine concentrations in surface water reached 41.2 ng/L [4]. In Costa Rica, surface water near coffee processing facilities contained caffeine levels as high as 1.1 mg/L [5]. In the European Union, effluents from wastewater treatment plants exhibited caffeine concentrations up to 3002 ng/L [6]. Similarly, in China, caffeine was detected in water bodies with average concentrations of 208 ng/L and 338 ng/L in the Guanting Reservoir and its upstream waters, respectively. Corresponding sediment concentrations were measured at 1430 ng/g and 1020 ng/g [7]. Moreover, over 25% of the rivers in the world are contaminated with pharmaceuticals, with caffeine ranking among the top three [8]. Recent studies have shown that caffeine exhibits teratogenic effects on zebrafish, which persisted even after a seven-day withdrawal period [9]. These findings collectively highlight the widespread contamination of aquatic ecosystems by caffeine and underscore the urgency of studying its environmental behavior.

The use of wastewater or contaminated surface water for irrigation can facilitate the transfer of caffeine into edible agricultural products, posing potential health risks [10,11]. For instance, Wu et al. [12] reported caffeine accumulation in the edible parts of celery, lettuce, and cabbage irrigated with reclaimed water containing 11 ng/L of caffeine, with concentrations reaching up to 0.17 μg/kg. Similarly, Goldstein et al. [13] found caffeine accumulation in cucumber and tomato fruits, with dry weight concentrations exceeding 1 ng/g. Consuming such contaminated products could have adverse health effects, as high caffeine intake is associated with elevated blood pressure, anxiety, insomnia, embryonic deformities, and dependence, particularly in vulnerable populations such as children, pregnant women, the elderly, and individuals with neurodegenerative conditions [14]. Caffeine absorption by plants from polluted water marks the initial step of its entry into the food chain, potentially endangering human health [15,16]. Therefore, understanding the accumulation and metabolism of exogenous caffeine in crops is crucial to assessing its risks to environmental quality, food safety, and human health.

Tea, one of the most worldwide consumed beverages, is traded internationally by over 150 countries. In 2023, the global tea market was valued at approximately USD 118.77 billion [17], reflecting its economic significance. With the increase in tea consumption, ensuring stricter safety standards has become essential. Tea plants, known for their ability to synthesize and metabolize caffeine, provide a unique model to study caffeine behavior in plants. Research shows that caffeine in tea leaves and roots undergoes demethylation to xanthine [18], which is further degraded into CO_2_ and NH_3_ through conventional purine metabolism [19]. At high caffeine concentrations, cytochrome P450 enzymes metabolize caffeine into theophylline and trimethyluric acid, while lower concentrations produce pseudoxanthine [20]. Studies on other plants, such as poplar and lettuce, have provided insights into caffeine metabolism. For example, Pierattini et al. [21] demonstrated that poplar trees metabolized exogenous caffeine into theobromine and theophylline, affecting endogenous compound levels. Similarly, Chuang et al. [22] reported that lettuce metabolized 54% of exogenous caffeine within six days via demethylation and oxidation, producing metabolites such as xanthine, 3-methylxanthine, theobromine, and theophylline. In conclusion, exogenous caffeine, introduced from external sources, undergoes demethylation and oxidation reactions in vegetables without further ring-opening mineralization, whereas endogenous caffeine, synthesized by the plant itself, follows intrinsic metabolic processes that include ring cleavage and mineralization into carbon dioxide. Despite these advances, the complete metabolic pathway of exogenous caffeine in tea plants remains unclear, with key intermediates yet to be identified [23]. Therefore, this study specifically aims to investigate how different tea plant varieties respond to exogenous caffeine, focusing on its accumulation, transport, and metabolic fate.

This study employs ^14^C isotope tracing to elucidate the targeted accumulation patterns and subcellular distribution of exogenous caffeine and its metabolites in a hydroponic tea seedling system. This study aims to fill this gap by examining how exogenous caffeine behaves in tea plants, thereby offering insights into the broader implications of caffeine contamination in agriculture.

## 2. Materials and Methods

### 2.1. Chemicals

The [8-^14^C]-labeled caffeine (radiochemical purity >98.5%, specific activity 53 mCi/mmol) was purchased from Moravek Biochemicals (Brea, CA, USA). The physical and chemical properties of caffeine are shown in Table S1. Unlabeled caffeine and xanthine (both with chemical purity >98%) were obtained from Absin Bioscience (Shanghai, China). Scintillation cocktail I was prepared by dissolving 0.5 g of 1,4-di(5-phenyloxazole-2-yl) (POPOP) and 7.0 g of 2,5-diphenyloxazole (PPO) in a solution of 400 mL 2-methoxyethanol and 600 mL dimethylbenzene. Scintillation cocktail II was similarly prepared, but with the addition of 175 mL ethanolamine and a modified solvent composition: 600 mL dimethylbenzene and 225 mL 2-methoxyethanol. The subcellular extraction buffer consisted of 50 mM Tris-HCl (pH 7.5), 250 mM sucrose, and 1.0 mM dithiothreitol.

### 2.2. Hydroponic Experiment

To explore whether different tea cultivars show variations in the uptake, transport, and metabolism of exogenous caffeine, healthy one-year-old tea seedlings (*Camellia sinensis* (L.) O. Kuntze) of the Longjing 43 and Jiaming No. 1 varieties were obtained from a specialty store in Xinchang (Shaoxing, China). Seedlings of each variety were cultivated in 250 mL flasks containing freshly prepared Hoagland nutrient solution (pH 6.0), with the flasks wrapped in aluminum foil to prevent light exposure to the roots. After two weeks of cultivation, 20 uniform and healthy seedlings with well-developed root systems from each variety were selected and transferred to 50 mL beakers. The plants were fixed, and the walls of the beakers were covered with aluminum foil. Growth conditions were maintained in a controlled chamber at 25 °C and 80% humidity, with illumination provided by LED lights (12,000 lx) under a 16 h light/8 h dark cycle.

### 2.3. Uptake and Translocation

To investigate caffeine uptake and translocation, caffeine at the concentration of 1.0 mg/L and 0.11 µCi/L was added to the Hoagland solution, and the seedlings were exposed to the treatment for 24, 48, 96, and 192 h. At each time point, three replicate hydroponic tea seedlings from each variety were collected. The seedlings were rinsed thoroughly with deionized water to remove any remaining nutrient solution. The roots were placed into pre-weighed centrifuge tubes, and the residual nutrient solution in the beakers was transferred to the same tubes. The beaker walls were rinsed with deionized water, and the rinse water was also added to the tubes. The total weight of each centrifuge tube with the nutrient solution was recorded, and the weight of the residual solution was calculated by subtracting the empty tube weight.

For radioactivity measurement, 1 mL of the nutrient solution was mixed with 12 mL of scintillation cocktail I and 1 mL of 2-methoxyethanol. The mixture was analyzed using a TriCarb 2910 liquid scintillation counter (LSC, Perkin-Elmer Inc., Downers Grove, IL, USA) to determine the specific radioactivity. After rinsing, the seedlings were air-dried to remove surface moisture, separated into aerial and root parts, and their fresh weights were recorded. The samples were then sealed in plastic bags and stored at −80 °C for subsequent analysis.

### 2.4. Subcellular Distribution

Buffers were prepared and pre-chilled in ice along with grinding jars. The aerial parts (shoots) and roots of fresh plant tissues were treated separately to chop and place into grinding jars, with two steel beads added. The tissues were then ground for 30 s at 60 Hz twice using a JXFSTPRP-24L grinder (Shanghai, China). The resulting homogenates were subjected to differential centrifugation at 4 °C to separate five subcellular components: cell wall, plastids, nuclei, mitochondria, and soluble fractions. The process involved the following steps: (1) The homogenized plant material was washed with buffer, vortexed thoroughly, and filtered through a 40-mesh filter into a 100 mL centrifuge tube. The residue retained on the filter was designated as the cell wall fraction; (2) the filtrate was centrifuged at 1500× *g* for 10 min (2500 g for 20 min for root samples) using a 5804R Eppendorf centrifuge (Hamburg, Germany), yielding the plastid fraction; (3) the supernatant from the previous step was centrifuged at 5000× *g* for 20 min to collect the nuclear fraction; (4) the subsequent supernatant was further centrifuged at 15,000× *g* for 30 min to isolate the mitochondrial fraction; (5) the final supernatant constituted the soluble fraction. For the soluble fraction, 0.5 mL of the supernatant was mixed with 0.5 mL deionized water, 12 mL scintillation cocktail I, and 1 mL of the solubilizing agent 2-methoxyethanol. The radioactivity was measured using a LSC to determine the specific radioactivity of the soluble components at each sampling time.

The pellets containing the cell wall, plastid, nuclear, and mitochondrial fractions were frozen at −20 °C and then lyophilized to constant weight. Approximately 0.1 g of each dried fraction was combusted in an OX-501 biological oxidizer (RJ Harvey Instruments Co., Hillsdale, NJ, USA) at high temperature (905 °C) and low temperature (680 °C) with a flow rate of 360 cc/min to ensure complete oxidation. The released ^14^CO_2_ was captured in 12 mL scintillation cocktail II with 1 mL of 2-methoxyethanol and analyzed for radioactivity on a LSC. After completing the subcellular distribution experiments, the total radioactivity of all subcellular components in the aerial parts and root parts of tea seedlings was calculated for each sampling time.

### 2.5. Pre-Treatment

Plant tissues that had been frozen at −80 °C were ground and extracted with methanol. Approximately 20 mL of methanol was added to each centrifuge tube containing the ground plant material, followed by vortexing for 1 h and ultrasonic extraction for 30 min. The samples were then centrifuged at 10,000× *g* for 10 min at 4 °C. This extraction process was repeated four times to ensure complete recovery of caffeine from both the aboveground and underground plant parts. After extraction, 0.5 mL of the extract was mixed with 12 mL of scintillation cocktail I to measure extractable residual caffeine, while the remaining residue was air-dried in a fume hood and subsequently oxidized in a biological oxidizer. The generated ^14^CO_2_ was trapped with 12 mL of scintillation cocktail II and analyzed by liquid scintillation counting to quantify bound residual caffeine.

The caffeine extract was concentrated using an EYELA N-1100 rotary evaporator (Tokyo, Japan). Residual concentrated extract in the round-bottom flask was washed with methanol into a glass bottle, and this process was repeated until approximately 10 mL of concentrated extract remained. The glass bottle was placed in an N-EVAP 111 nitrogen evaporator (Organomation, MA, USA), where the extract was gently dried under a nitrogen stream until the volume was reduced to 1 mL. The concentrated extract was then purified using a ProElut CARB/PSA column (500 mg/6 mL) to remove chlorophyll and metal ions. The column was pre-activated with 5 mL of deionized water and 5 mL of methanol. After activation, the concentrated extract was applied to the column, and eluates from three replicate samples of the same variety and tissue type were combined into a single centrifuge tube. Each glass bottle was rinsed with 5 mL of methanol, and the wash was collected into the corresponding centrifuge tube. The combined eluate was dried under a nitrogen stream, re-dissolved in 1.0 mL of methanol, filtered through a 0.22 μm JT membrane (Φ13 mm, Tianjin, China), and transferred into a 2 mL sample vial to obtain the final caffeine and its metabolite extract.

### 2.6. LC–QTOF-MS Analysis

Extracts were separated using an Agilent 1260 liquid chromatography system (Agilent Co., CA, USA) with a Diamonsil C_18_ column (5 µm, 250 × 4.6 mm, DiKMA, CA, USA). The separation was performed at a flow rate of 1 mL/min and a column temperature of 40 °C, with an injection volume of 20 µL. The mobile phase comprised 0.1% formic acid in deionized water (A) and 0.1% formic acid in acetonitrile (B). The gradient elution program was as follows: 0–5 min, 5% B; 5–30 min, 5–100% B; 30–33 min, 100% B; 33–34 min, 100–5% B; and 34–40 min, 5% B. After separation, fractions were collected at one-minute intervals, yielding 40 fractions per sample. The radioactivity of each fraction was measured using an LSC. Standard caffeine solutions (2, 4, 6, 8, and 10 mg/L) were prepared and analyzed under the same chromatographic conditions to construct a calibration curve, which was used to determine the retention time of caffeine.

For mass spectrometry analysis, the QTOF-MS system operated in positive electrospray ionization (ESI) mode over a mass range of m/z 50–1500. Key parameters included a capillary voltage of 4000 V, a cone voltage of 65 V, a drying gas temperature of 250 °C, and a flow rate of 8 L/min. The fragmentor and skimmer voltages were set at 175 V and 19 V, respectively, with MS/MS collision energy ramped from 5 to 50 V. Data acquisition and processing were conducted using Agilent MassHunter Workstation Software (version B.06.00).

### 2.7. Statistical Analysis

Statistical analyses were conducted using SPSS 20.0.0, with ANOVA employed to assess differences at a significance level of *p* < 0.05. Graphs were created using Origin Pro 9.0.0, and results are expressed as mean ± standard error (*n* = 3).

## 3. Results and Discussion

### 3.1. Absorption and Distribution Characteristics of ^14^C-Caffeine in Hydroponic Tea Seedling Systems

**Figure 1:**
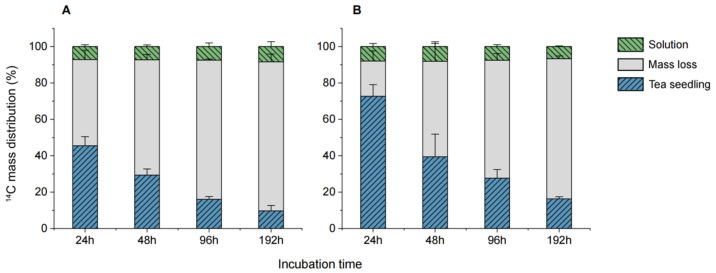
Percentage distribution of ^14^C mass in hydroponic tea seedling systems. (**A**) represents Longjing 43, and (**B**) represents Jiaming No. 1.

To investigate the absorption and accumulation patterns of caffeine in hydroponic systems for the tea seedlings of Longjing 43 and Jiaming No. 1, the experimental system was divided into three parts: plant, water body, and “loss” segment. As shown in Figure 1, ^14^C was detected in both tea seedling varieties at all sampling times, indicating their ability to absorb and accumulate exogenous caffeine from the nutrient solution.

The ^14^C content in the nutrient solution decreased progressively over time. At 192 h, the remaining ^14^C content in the nutrient solution was 9.7% for Longjing 43 and 16.3% for Jiaming No. 1. Conversely, the ^14^C content in the plants remained relatively stable over the cultivation period. By 192 h, the total ^14^C proportion in Longjing 43 showed an increase, ranging from 7.16% to 8.42%. In Jiaming No. 1, the total ^14^C proportion first increased and then decreased, peaking at 48 h with a range of 6.64–8.13%. These trends suggest distinct uptake and distribution patterns between the two tea varieties over time.

The ^14^C mass balance further illustrated differences in recovery rates. In the Longjing 43 system, the recovery rate ranged from 18.13% to 52.66%, while in the Jiaming No. 1 system, it ranged from 22.97% to 80.67%. By the final sampling point, approximately 82% of ^14^C in the Longjing 43 system and 77% in the Jiaming No. 1 system was categorized as “lost”. Similar trends were observed in studies on lettuce, where caffeine mass balance fell below 50% after 144 h, indicating a “loss” of approximately 60% [24]. Chen et al. [25] reported 50–80% losses in hydroponic systems involving Chinese flowering cabbage and water spinach, attributing the losses to caffeine mineralization into carbon dioxide released into the air. Earlier research suggests that caffeine undergoes metabolic degradation in both plants and microorganisms, producing carbon dioxide [26]. Li et al. [27] investigated phenanthrene in hydroponic wheat systems and found mineralization generated 12–16% carbon dioxide, while metabolism produced 4–5% volatile organic compounds. In the current study, the use of brown glass culture bottles wrapped in aluminum foil effectively minimized photodegradation of caffeine. Therefore, the “lost” ^14^C likely underwent microbial metabolism in the nutrient solution or was absorbed by the plants and subsequently metabolized or mineralized into volatile gases released into the air.

### 3.2. Accumulation of ^14^C-Caffeine in Hydroponic Tea Seedlings

**Figure 2:**
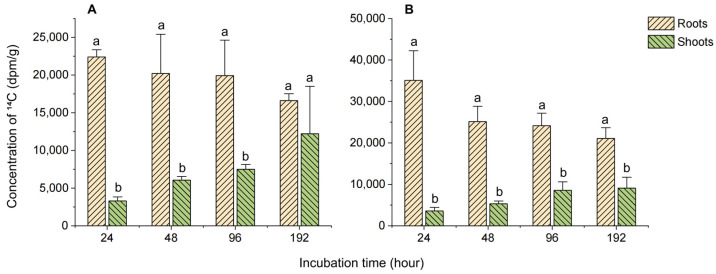
Dynamic concentration of ^14^C in different tissues of hydroponically grown tea seedlings. (**A**) Longjing 43 hydroponic system; (**B**) Jiaming No. 1 hydroponic system. Different letters (a,b) above the histograms indicate significant differences between tissues at the same time point (*p* < 0.05).

The analysis of caffeine distribution in the hydroponic tea seedling system reveals that both Longjing 43 and Jiaming No. 1 tea plants effectively absorbed and accumulated exogenous caffeine from the water. The distribution trends of ^14^C-caffeine and its metabolites in plant tissues were quantified, as shown in Figure 2. Over the 192 h cultivation period, ^14^C-caffeine and its metabolites were predominantly absorbed by the roots and subsequently transported to the aerial parts of the seedlings. The results demonstrate that the ^14^C concentration in the roots of both tea seedling varieties decreased over time, while it increased in the aerial parts. This finding indicates that caffeine absorption by the roots is followed by delayed upward transport through transpiration. Consequently, the peak concentration in the aerial parts occurred later than in the roots. Notably, except for the Longjing 43 group at 192 h, the ^14^C concentration of in the roots consistently exceeded that in the aerial parts for both varieties. At the final sampling point of 192 h, the ^14^C concentrations in the roots and aerial parts of Longjing 43 were 16,610 dpm/g and 12,210 dpm/g, respectively, while those of Jiaming No. 1 were 21,075 dpm/g and 9092 dpm/g.

The high water solubility and small molecular size of caffeine facilitate its transport via the transpiration stream, leading to its accumulation in tissues with high transpiration rates. For instance, in crops like cabbage, water spinach, or lettuce that lack endogenous caffeine, caffeine accumulates preferentially in basal leaves with larger leaf areas, lower water potential, and higher transpiration rates [22,25,28]. However, in tea plants, two factors appear to influence caffeine translocation: (1) endogenous caffeine in tea leaves may create a concentration gradient that limits the upward movement of exogenous caffeine, and (2) caffeine metabolism in tea plants, particularly in older leaves, is closely tied to nucleic acid and protein catabolism, where degradation of these molecules promotes caffeine breakdown [23]. Consequently, the higher ^14^C concentration in the roots compared to the aerial parts observed in this study may be attributed to endogenous caffeine production and metabolism in tea plants.

### 3.3. Root Concentration Factor and Translocation Factor

The root concentration factor (RCF) represents the ratio of organic pollutant concentration in plant roots to that in the environment, serving as a critical indicator of pollutant accumulation in plants. The formula for calculating BCF is as follows:

(1) $$\mathrm{RCF}\;({\mathrm{mL}\;g}^{-1})=\frac{\begin{array}{c} Concentration\;of\;pollutants\;in\;roots\;(\mu g/g) \end{array}}{\mathrm{Initial}\;\mathrm{concentration}\;\mathrm{of}\;\mathrm{pollutants}\;\mathrm{in}\;\mathrm{nutrient}\;\mathrm{solution}\;(\mu g/\mathrm{mL})}$$

The dynamic changes in the RCF of Longjing 43 and Jiaming No. 1 tea seedlings over different exposure times are shown in Table 1. Results indicate that RCF values for both varieties decreased with prolonged exposure, with Longjing 43 ranging from 1.35 to 1.82 mL/g and Jiaming No. 1 ranging from 1.72 to 2.85 mL/g. At all sampling points, Jiaming No. 1 consistently exhibited higher RCF values, with significant differences observed at 24 h and 192 h, suggesting greater accumulation of caffeine and its metabolites in its roots. Comparison with other organic pollutants reveals that highly hydrophobic compounds, such as triclocarban, triclosan, and fluoxetine, tend to accumulate in plant roots, with bio-concentration factor values reaching 111−840 L/kg, while compounds like meprobamate, atorvastatin, diclofenac, and acetaminophen exhibit minimal root accumulation, with bio-concentration factors below 5 L/kg [29,30,31,32,33]. Certain pollutants, including carbamazepine and diazepam, display high mobility within plants, leading to significant bio-concentration in leaves. Environmental factors also play a crucial role in pollutant behavior in plants [34]. Goldstein et al. [13] demonstrated that physiological traits of crops, physicochemical properties of pharmaceuticals, soil characteristics, and water quality collectively influence the absorption, transport, and accumulation of pharmaceuticals in plant tissues under wastewater irrigation. For example, physiological differences between fruits result in higher caffeine concentrations in cucumbers irrigated with reclaimed wastewater compared to tomatoes. The observed differences in RCF values between Longjing 43 and Jiaming No. 1 tea seedlings in this study are likely attributed to the physiological characteristics of these tea varieties, which may regulate their capacity for caffeine uptake and accumulation.

The translocation of organic pollutants within plants is quantified by the translocation factor (TF), which is defined as the ratio of pollutant concentration in the shoots to that in the roots, as calculated by Equation (2).

(2) $$\mathrm{TF}=\frac{\mathrm{Concentration}\;\mathrm{of}\;\mathrm{pollutants}\;\mathrm{in}\;\mathrm{shoots}}{\mathrm{Concentration}\;\mathrm{of}\;\mathrm{pollutants}\;\mathrm{in}\;\mathrm{roots}}$$

Variations in TF values for hydroponically grown Longjing 43 and Jiaming No. 1 tea seedlings at different sampling times are summarized in Table 1. Both varieties exhibited increasing TF values with prolonged exposure, ranging from 0.18 to 0.59 for Longjing 43 and 0.07 to 0.46 for Jiaming No. 1. Despite the upward trend, average TF values remained below 1 after 192 h of exposure, indicating limited translocation of pollutants from roots to shoots. At the same sampling times, Longjing 43 showed slightly higher average TF values than Jiaming No. 1, though the differences were not statistically significant. This suggests comparable capacities for chemical translocation between the two varieties. The movement of organic pollutants within plants is influenced by factors such as their dissociation properties, hydrophobicity, and the plant’s transpiration activity. Studies have established a linear relationship between the ease of root-to-shoot translocation of organic pollutants and their octanol–water partition coefficient (log *K*_OW_), with hydrophobic compounds tending to accumulate in roots and hydrophilic compounds being more readily transported to shoots via transpiration [35]. Furthermore, organic compounds with moderate polarity (log *D*_OW_ between 0.5 and 3) exhibit higher translocation rates to aerial parts of plants [33]. The role of transpiration in the accumulation and transport of acidic, basic, and neutral organic pollutants within plants [36]. The waxy cuticle of tea leaves, compared to the succulent leaves of cabbage or water spinach, effectively reduces water loss through transpiration [37,38]. This character slows the upward translocation of caffeine and contributes to its higher accumulation in the roots.

### 3.4. Subcellular Distribution of ^14^C-Caffeine in Hydroponic Tea Seedlings Systems

**Figure 3:**
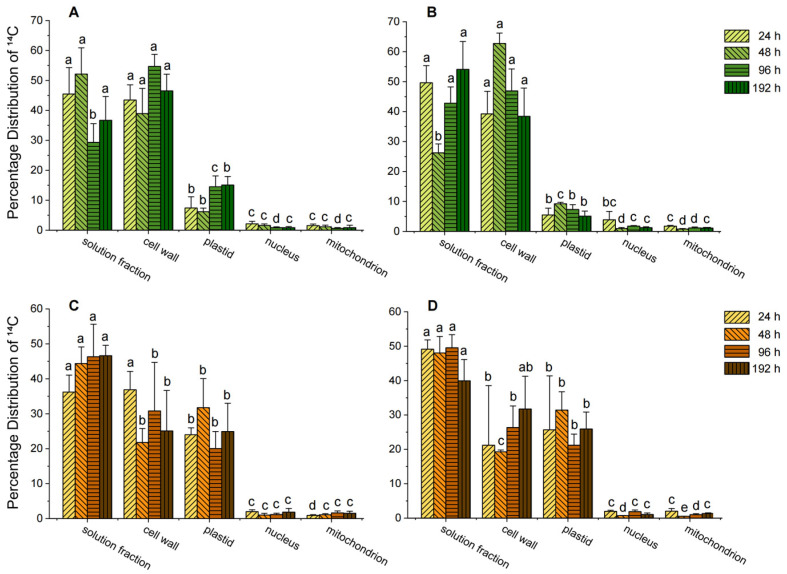
Subcellular distribution of ^14^C-caffeine and its metabolites in different components of tea seedlings. (**A**,**B**) represent the aerial parts of Longjing 43 and Jiaming No. 1 seedlings, while (**C**,**D**) represent their roots; a–e indicate differences among different cellular components at the same time point.

This study examined the subcellular distribution of ^14^C-caffeine in the shoots and roots of tea seedlings under hydroponic conditions to elucidate the mechanisms of organic pollutant uptake and transport in plants. As shown in Figure 3, cells in both shoots and roots were divided into five components: cell wall, plastids, nucleus, mitochondria, and soluble fraction. The presence of ^14^C in all these components demonstrates that caffeine and its metabolites could penetrate cell walls and membranes, enabling transport into various organelles.

In root cells, ^14^C-caffeine and its metabolites were predominantly distributed in the soluble fraction, cell wall, and plastids. For Longjing 43 tea seedlings, the soluble fraction accounted for 36.2–46.6%, the cell wall for 21.8–36.9%, and plastids for 20.7–31.7%. In Jiaming No. 1 tea seedlings, the soluble fraction represented 39.9–49.5%, the cell wall 19.3–31.7%, and plastids 21.2–31.4%. Smaller amounts were detected in the nucleus and mitochondria. Over time, the proportion of ^14^C in the soluble fraction of Longjing 43 root cells increased, while it decreased in the cell wall, suggesting that caffeine and its metabolites initially binds to the cell wall and subsequently transfers to the soluble fraction (Figure 3C). Conversely, in Jiaming No. 1, the proportion of ^14^C in the soluble fraction decreased while increasing in the cell wall, indicating potential storage of caffeine and its metabolites in the cell wall (Figure 3D). Other subcellular components exhibited fluctuating ^14^C levels without clear trends.

In shoot cells, ^14^C-caffeine and its metabolites were mainly distributed in the soluble fraction and cell wall, with lesser amounts in plastids. For Longjing 43 seedlings, the soluble fraction accounted for 29.31–52.15%, and the cell wall 38.9–54.7% (Figure 3A). For Jiaming No. 1, the soluble fraction contributed 26.2–54.1%, and the cell wall 38.4–62.7% (Figure 3B). Minimal amounts were detected in the nucleus and mitochondria. Over time, the subcellular distribution in the shoots of both varieties displayed fluctuations but followed trends similar to those observed in the roots, with caffeine predominantly residing in the soluble fraction and cell wall.

Overall, the subcellular distribution results identify the soluble fraction as the primary reservoir for ^14^C-caffeine and its metabolites in tea seedlings. This may be attributed to the low adsorption affinity of caffeine by plant roots and its transport to leaves via transpiration flow [24]. For highly water-soluble pollutants, such as pharmaceuticals and personal care products, the aqueous phase of plant cells often serves as their primary reservoir [25,39]. In this study, caffeine, a hydrophilic compound with a log *K*_OW_ of −0.07, exhibited similar behavior, with a significant proportion accumulating in the soluble fraction of plant cells.

### 3.5. Metabolism of Caffeine in the Tea Seedling

**Figure 4:**
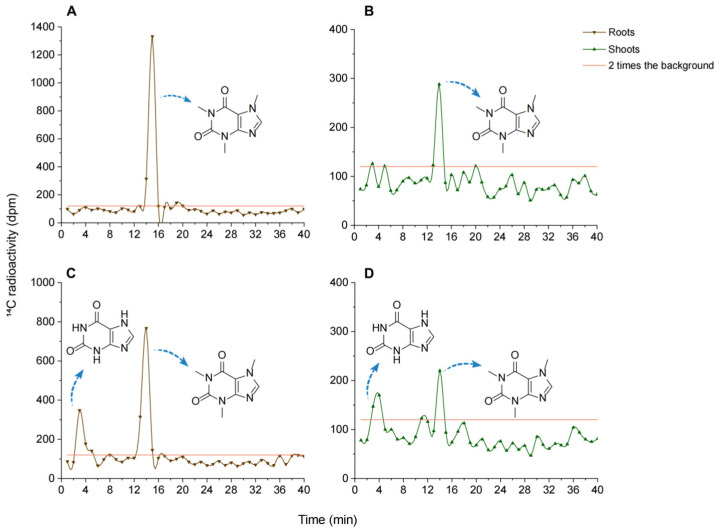
Radioactive chromatograms of extracts from the roots and shoots of Longjing 43 (**A**,**B**) and Jiaming No. 1 (**C**,**D**) tea seedlings.

The HPLC effluent was collected using a fraction collector, and two distinct radioactive peaks were quantified with an LSC detector. The radioactive chromatogram, presented in Figure 4, shows retention times of 3–5 min and 12–15 min. These retention times were compared with those in the LC–QTOF-MS mass spectrum to identify the compounds. After 192 h of exposure to caffeine, extracts from the roots and shoots of Longjing 43 and Jiaming No. 1 seedlings were analyzed using liquid chromatography, and the ^14^C-radioactivity in the effluent for each minute was recorded (Figure 4). Peaks exceeding twice the background radioactivity level (~120 dpm) were identified as ^14^C-caffeine or its metabolites. Based on the standard curve and retention time of caffeine, the compound at 12–15 min was identified as the caffeine parent (Figure S1). In Jiaming No. 1 seedlings, an additional metabolite peak with a retention time of 3–5 min was detected in both root and shoot extracts. This peak, significantly exceeding twice the background level, was identified as xanthine according to its retention time and radioactive peak intensity. The structure of xanthine and caffeine was further confirmed by comparison with standards (Figures S2–S3).

Previous studies indicate that caffeine undergoes metabolism in plants, often producing intermediates or end products with bioactive properties [22]. For example, exogenous caffeine metabolism in poplar generated theobromine and theophylline [21]. In lettuce exposed to exogenous caffeine, demethylation and oxidation reactions were identified, leading to metabolites such as theobromine, hypoxanthine/theophylline, 3/7-methylxanthine, and xanthine [22]. Furthermore, they suggested that 62.1% of the caffeine in lettuce shoots is metabolized into CO_2_. In plants with endogenous caffeine, such as tea and coffee trees, the primary metabolites are theophylline and theobromine, which are further converted into 3-methylxanthine and xanthine, eventually breaking down into CO_2_ and NH_3_ [40,41,42]. In Longjing 43 seedlings, caffeine decomposed rapidly without detectable intermediates. Conversely, in Jiaming No. 1 seedlings, caffeine metabolism produced xanthine, whose slower degradation limited the mineralization rate of caffeine into CO_2_. This metabolic difference likely explains the slower rate of caffeine mineralization into CO_2_ observed in Jiaming No. 1 compared to Longjing 43 seedlings. Consequently, Jiaming No. 1 retained higher ^14^C levels in the nutrient solution under identical exposure conditions.

## 4. Conclusions

This study systematically investigated the absorption, transport, and cellular compartmentalization of caffeine, a typical organic pollutant, in tea seedlings. The results demonstrate that tea seedlings effectively absorbed and enriched exogenous caffeine from the water. Over the cultivation period, the proportion of ^14^C activity in the water medium decreased, while its content in the plants remained stable. After 192 h, ^14^C-caffeine and its metabolites were transported to the shoots but predominantly accumulated in the roots. At the subcellular level, ^14^C was primarily distributed in the soluble fractions, cell walls, and plastids within the roots, whereas in the shoots, it was mainly found in the soluble fractions and cell walls. Notably, in Longjing 43 tea seedlings, caffeine was detected predominantly as the parent compound, while in Jiaming No. 1 seedlings, both the parent compound and the metabolite xanthine were identified. The study provides novel insights into the varietal differences in caffeine metabolism and the distribution of exogenous caffeine in tea plants, thereby contributing to a more comprehensive evaluation of its environmental safety. While caffeine contamination in the environment is a concern, the concentrations observed in this study may not pose an immediate risk. However, further studies are necessary to assess the long-term environmental impact and potential risks at the levels of microgram or nanogram.

## Figures and Tables

**Table 1:** Root concentration factor (RCF) and translocation factor (TF) of ^14^C in hydroponic tea seedlings system.

Variety	Time (Hour)	RCF Value (mL/g)	TF Value
Longjing 43	24	1.82 ± 0.08 b	0.15 ± 0.03 a
	48	1.65 ± 0.42 a	0.31 ± 0.06 a
	96	1.62 ± 0.38 a	0.39 ± 0.11 a
	192	1.35 ± 0.07 b	0.74 ± 0.4 a
Jiaming No.1	24	2.85 ± 0.58 a	0.11 ± 0.04 a
	48	2.05 ± 0.3 a	0.21 ± 0.01 a
	96	1.96 ± 0.25 a	0.35 ± 0.05 a
	192	1.72 ± 0.21 a	0.44 ± 0.14 a

Note: Different letters (a,b) indicate significant differences between varieties at the same time point (*p* < 0.05).

## Data Availability

The data are available from the corresponding author.

## Supplementary Materials

The following supporting information can be downloaded at: https://www.mdpi.com/article/10.3390/biology14020177/s1, Figure S1: Calibration curve of caffeine; Figure S2: Liquid chromatogram and QTOF-MS spectrum of xanthine; Figure S3: Liquid chromatogram and QTOF-MS spectrum of caffeine; Table S1: Physiochemical properties of caffeine.

## Abbreviations

The following abbreviations are used in this manuscript:

POPOP	1,4-di(5-phenyloxazole-2-yl)
PPO	5-diphenyloxazole
LSC	Liquid scintillation counter
LC–QTOF-MS	Liquid chromatograph system coupled to an quadrupole time of flight mass spectrometer
BCF	Bio-concentration factor
TF	Linear dichroism
