# Supplementary material for: Varietal Differences in the Environmental Behavior of 14C-Caffeine in Tea Plants: Accumulation, Subcellular Distribution, and Metabolism
Source: Biology (Basel). 2025 Feb 10;14(2):177. doi: 10.3390/biology14020177 (PMC11851553; doi:10.3390/biology14020177)
Supplement: Supplementary file 1 [file biology-14-00177-s001.zip › biology-3441209-supplementary.pdf]

Supplementary Materials for

Varietal Differences in the Environmental Behavior of ^{14}C-Caffeine in Tea Plants: Accumulation, Subcellular Distribution and metabolism

Yan Chen *et al.*

Corresponding author: Xinqiang Zheng, xqzheng@zju.edu.cn

This file includes:

Figures S1 to S3

Table S1

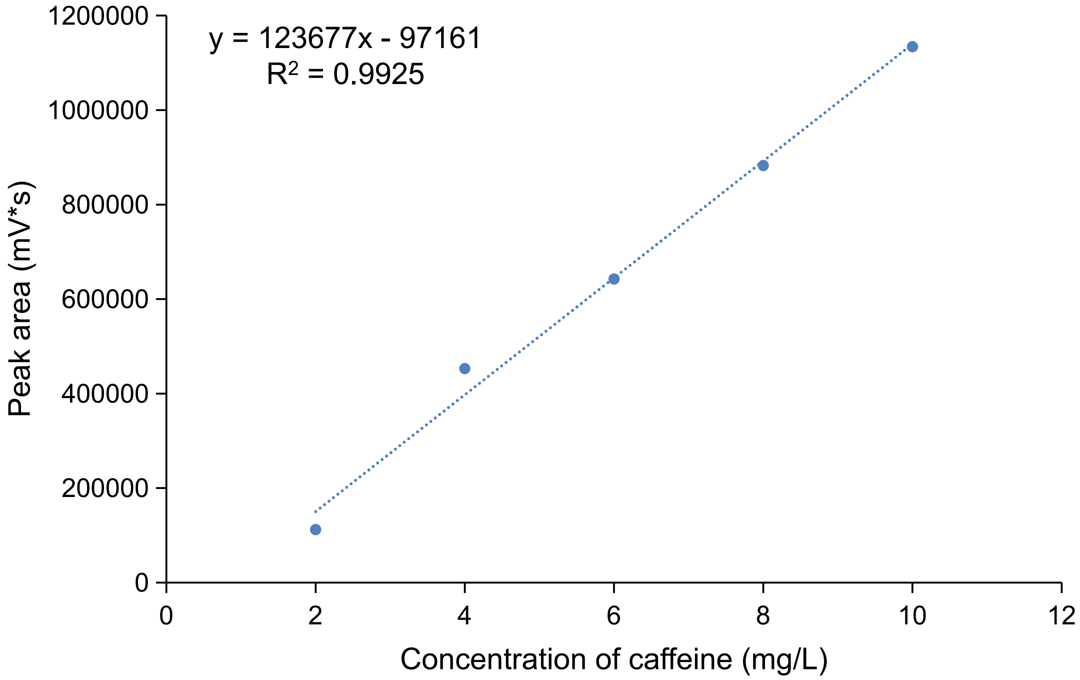

Figure S1 Calibration curve of caffeine.

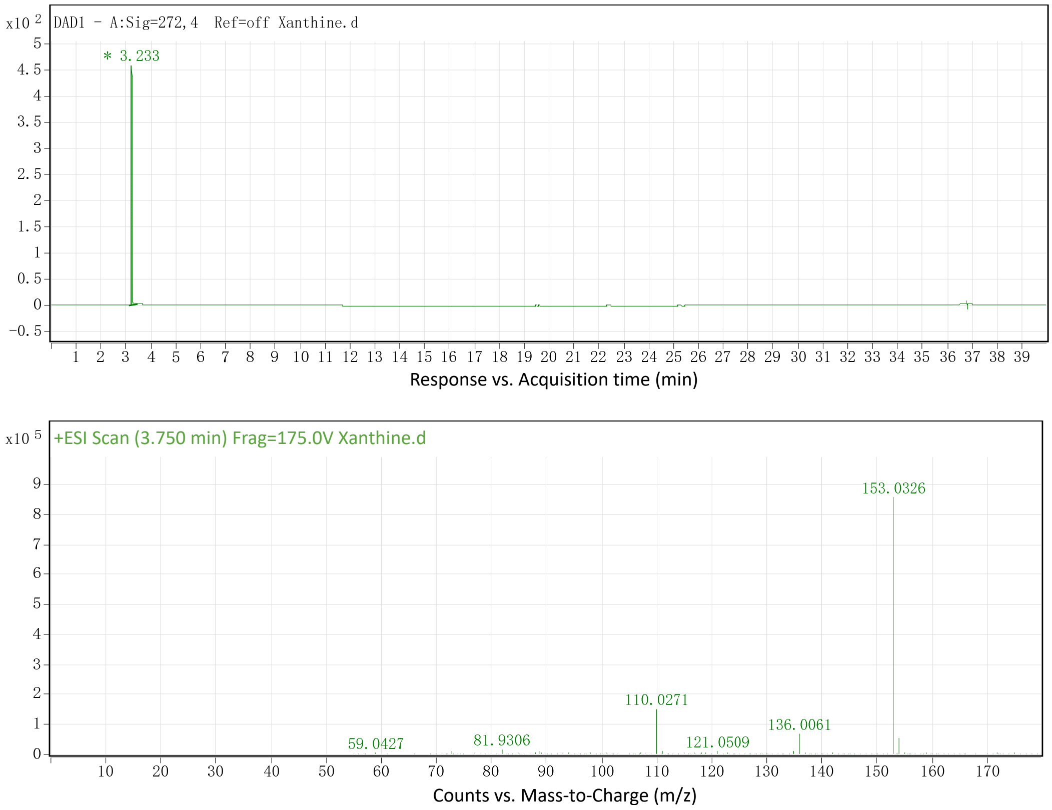

Figure S2 Liquid chromatogram and QTOF-MS spectrum of xanthine.

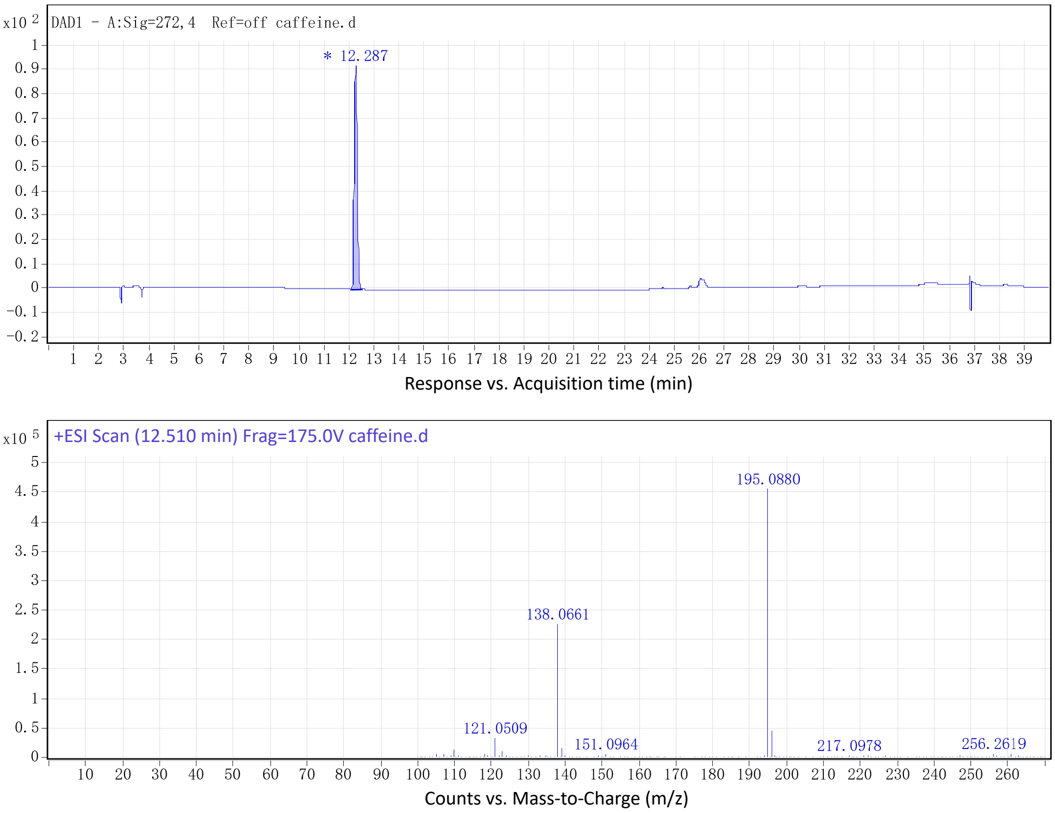

Figure S3 Liquid chromatogram and QTOF-MS spectrum of caffeine.

Table S1 Physiochemical properties of caffeine.

Property	Caffeine
Molecular Structure	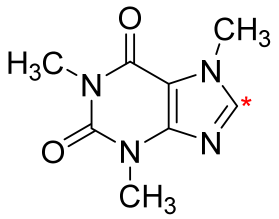
Molecular Formula	$C_8H_{10}N_4O_2$
Relative Molecular Mass	194.19
log K_{OW}	−0.07
Water Solubility (mg L^{-1}) [°C]	21600 [25°C]
Boiling Point (°C)	178

*Note: The ^{14}C labeling site is indicated with an asterisk *.*
